# Development of an Immunochromatographic Strip Test for Rapid Detection of Ciprofloxacin in Milk Samples

**DOI:** 10.3390/s140916785

**Published:** 2014-09-10

**Authors:** Liqiang Liu, Liju Luo, Steven Suryoprabowo, Juan Peng, Hua Kuang, Chuanlai Xu

**Affiliations:** State Key Lab of Food Science and Technology, School of Food Science and Technology, Jiangnan University, Wuxi 214122, China; E-Mails: raxray@gmail.com (L.L.); 1049628667@qq.com (L.L.); steven_090287@yahoo.co.id (S.S.); pengjuan2016@163.com (J.P.); khecho@163.com (H.K.); xcl@jiangnan.edu.cn (C.X.)

**Keywords:** ciprofloxacin, immunochromatographic assay, test strip, monoclonal antibody

## Abstract

A rapid, simple, and sensitive immunochromatographic test strip has been developed for testing residues of ciprofloxacin (CIP). A specific and sensitive monoclonal antibody (mAb) for CIP was generated by immunizing BALB/c mice with well-characterized CIP-Keyhole limpet haemocyanin. Under the optimized conditions, the cut-off limits of test strips for CIP were found to be 5 ng/mL in phosphate-buffered saline and 2.5 ng/mL in milk samples. Each test can be evaluated within 3 min. The cross-reactivities of the CIP test strip to enrofloxacin (ENR), norfloxacin (NOR), nadifloxacin (NDF), danofloxacin (DANO), pefloxacin (PEX), lomefloxacin (LOME), enoxacin (ENO), and sarafloxacin (SAR) were 71.4%, 71.4%, 66%, 50%, 33%, 20%, 12.5%, and 6.25%, respectively. The data indicate that the method is sensitive, specific, and has the advantages of simplicity and speed, therefore, this test strip is a useful screening method for the detection of CIP residues in milk samples.

## 1. Introduction

Fluoroquinolones (FQs) are the most important group of synthetic antimicrobials, which are widely used in veterinary and aquatic medicine. FQs act by inhibiting DNA synthesis by targeting the essential inhibition of bacterial enzymes DNA gyrase and topoisomerase IV [1,2] Infectious diseases are important key factors affecting the profitability of livestock industries. The presence of antimicrobial drug residues in food carries potential risk by selection of resistant pathogenic organisms and causes adverse effects on intestinal microflora, which could decrease the quality of animal products. In the mid-1980s and 1990s, FQs were introduced for human use in Europe and the United States and approved for livestock treatment [3] and quickly become the most commonly prescribed antibiotics.

**Figure 1.**
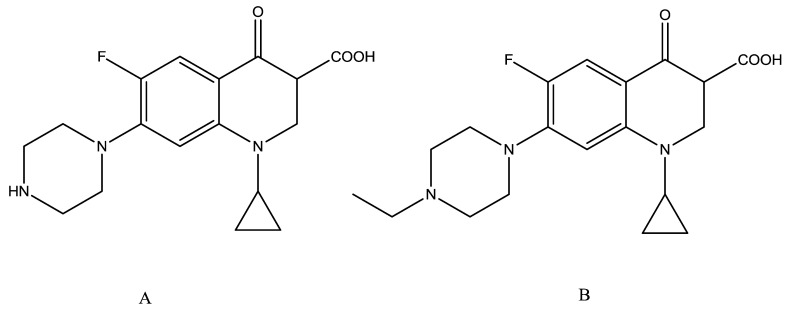
Chemical structure of: (**A**) ciprofloxacin; (**B**) enrofloxacin.

Ciprofloxacin (CIP, Figure 1A) is the most commonly used FQ for the treatment of urinary tract infections. The effects of CIP residues are lower respiratory tract infections, nosocomial pneumonia, skin infections, and chronic bacterial prostatitis [4–7]. The widespread use and possibility of abuse or misuse of FQs in agriculture and aquaculture has resulted in the potential presence of residues of these compounds in foodstuffs of animal origin, which has given rise to public health concerns over their toxic effects, development of resistant strains of bacteria, and allergic hypersensitivity reactions [8–10]. Because of the concerns about drug residues entering the food chain and contributing to bacterial resistance, the use of antibiotics in livestock is strictly regulated and maximum residue levels (MRLs) have been established. In the European Union MRLs has been set for enrofloxacin and ciprofloxacin in milk (100 ng/mL) by Council Regulation EEC/2377/90. Consequently, there is a growing demand for easy, rapid, and economical methods to detect and manage FQ residues in foodstuffs.

Microbial assays to determine CIP in food products are usually simple and inexpensive, but these methods are time consuming, and lack specificity [11]. Instrumental methods, including liquid chromatography-mass spectrometry (LC-MS) and high-performance liquid chromatography (HPLC), which LOD is approximately 2 μg/kg, [12–14] are the most widely used method to detect FQ residues. Even though these methods are sensitive and highly specific, they require expensive equipment, large volumes of solvents, highly trained individuals for operating complicated instruments, and are time consuming because of the needed sample cleanup processes, and generally are not suitable for use in the field.

Analytical methods with increased throughput, reduced environment impact, and involving rapid and inexpensive screening methods are required. An enzyme-linked immunosorbent assay (ELISA) based on a monoclonal antibody (mAb) has been successfully developed for the detection of ciprofloxacin residues with calculated LOD values of 0.06 ng/mL for nine kind of FQs [4] or for two kind different quinolone analogues with a detection limit of 0.32 ng/m · L [15], and Jiang *et al.* also developed a monoclonal antibody against small hapten-ciprofloxacin with a detection limit of 1.56 ng/mL that had cross-reactivity with enrofloxacin [16].

Lateral-flow immunochromatographic assays are increasingly popular as a diagnostic tool for detecting drug residues because of their simplicity, speed, specificity and sensitivity. Compared with ELISA, lateral-flow immunochromatographic assays have advantages such as all the reagents are included in the strip and the results can be obtained within 5–10 min [17–19]. A lateral-flow immunochromatographic assays for difloxacin detection with 0.24% cross-reactivity towards danofloxacin and no cross-reactivity towards other related compounds has been described by Zhi *et al.* [20] and an immunochromatographic strip for the detection of enrofloxacin residues with a lower detection limit was 100 ng/mL has been described by Kim *et al.* [21].

The principle of immunochromatographic lateral-flow testing has broad applicability (rapid, simple, and effective) and to the best of our knowledge, such an immunochromatographic lateral-flow test device for the detection of CIP residues has not been previously reported. The aim of this study was: (a) to develop an immunochromatographic lateral-flow test strip for the detection of ciprofloxacin in milk samples; (b) to detect other fluoroquinolones (FQs) by using an anti-ciprofloxacin antibody.

## 2. Material and Methods

### 2.1. Chemicals and Reagents

CIP, enrofloxacin (ENR), norfloxacin (NOR), nadifloxacin (NDF), danofloxacin (DANO), pefloxacin (PFX), lomefloxacin (LOME), enoxacin (ENO), and sarafloxacin (SAR) were purchased from J&K Scientific (Shanghai, China). Complete Freund's adjuvant (FCA), incomplete Freund's adjuvant (FIA), and enzyme immunoassay-grade horseradish peroxidase-labeled goat anti-mouse immunoglobulin were obtained from Sigma (St. Louis, MO, USA). Gelatin was obtained from Beijing Biodee Biotechnology Co., Ltd. (Beijing, China). Tetramethylbenzidine (TMB) and horseradish peroxidase (HRP) were purchased from Aladdin Chemistry Co., Ltd. (Shanghai, China). All reagents for cell fusion were gotten from Sunshine Biotechnology Co., Ltd. (Nanjing, China). Bovine serum albumin (BSA), ovalbumin (OVA) and keyhole limpet hemocyanin (KLH) were obtained from Solarbio Science & Technology, Co, Ltd, (Beijing, China). Other reagents and chemicals were obtained from the National Pharmaceutical Group Chemical Reagent Co., Ltd. (Shanghai, China).

Nitrocellulose high-flow plus membranes (Pura-bind RP) were from Whatman-Xinhua Filter Paper Co., Ltd. (Hangzhou, China). The glass fibre membrane (CB-SB08) used for sample pad, the polyvinylchloride (PVC) backing material and the absorbance pad (SX18) were supplied by Goldbio Tech Co., Ltd. (Shanghai, China). A BioDot TSR3000 Membrane Strip Reader (Gene Company Limited, Shanghai Branch, Shanghai (China) was used to test the color intensities of colloidal gold on the test zone.

### 2.2. Preparation and Characterisation of Monoclonal Anti-Ciprofloxacin (CIP) Antibody

CIP was conjugated to BSA and KLH using the active ester method [4]. Briefly, A mixture CIP (5 mg), carboxyl-reactive carbodiimide cross linker (EDC, 8.67 mg), and N-hydroxysuccinimide-(NHS, 5.21 mg) were added to DMF (1 mL) and incubated for 24 h in a dark chamber (solution 1). KLH (14 mg) or BSA (33.5 mg) was mixed with 0.01 M PBS (3 mL, solution 2). Solution 1 was slowly added to solution 2 with stirring and the mixture was stirred continuously for 8 h at room temperature. The resulting supernatant was dialyzed against PBS for 2 days with four changes of PBS solution during this period to remove free CIP. Ultraviolet absorption was used to check the conjugation of protein and CIP.

For the production of monoclonal antibodies (mAbs), female BALB/c mice (6–8 weeks old) were subcutaneously immunized with the CIP-KLH conjugate. FCA was used for the first immunization and FIA was used in the subsequent boost injection. Mice were immunized every three weeks with 100 μg for the first immunization and dose of 50 μg for the remainder. Blood samples from the immunized mice were measured by ELISA, and the mouse with the highest titer was sacrificed and its splenocytes were fused with Sp 2/0 murine myeloma cells, after which hybridomas were screened using an indirect ELISA. The selected hybridoma cells were expanded and injected into BALB/c mice to produce the monoclonal antibody (mAb) [22]. Ascites was harvested and purified using the caprylic acid-ammonium sulfate precipitation method [23]. The purified antibody solution was divided into small aliquots and stored at −20 °C until further use.

### 2.3. Development of Lateral-Flow Test Device

#### 2.3.1. Preparation of Colloidal Gold Particles

Based on Sun *et al.* [18], all solvents were prepared with Millipore-Q water and then filtered through a transfer membrane (0.22 μm). Fifty millilitre of a 0.1 g/L chlorauric acid solution was heated to boiling under constant stirring (100 rpm), and then, 1% w/v trisodium citrate solution (2.0 mL) was added. The mixture was stirred for 6 min. The color of the solution turned wine-red and then was cooled at room temperature and stored at 4 °C for future use. Transmission electron microscopy (TEM) data showed that the gold nanoparticles had a nearly uniform particle size of 30 nm. The UV-visible spectra characterized the maximum absorbance peak at 530 nm.

#### 2.3.2. Preparation of Colloidal Gold Labeled mAb

The anti-CIP monoclonal antibody (mAb) [4] with an IC_50_ value of 0.57 ng/mL was prepared before. The pH of the 10 mL colloidal gold solution for conjuction was adjusted to pH 7.0 with 0.1 M K_2_CO_3_. Briefly, mAb (0.25 mL) was added to the solution dropwise, and 50 min later, 0.5% (w/v) casein (1 mL) was added and the mixture stirred for 2 h. The products were centrifuged for 50 min in 7000 rpm to remove the gold aggregates and then the red supernatant was centrifuged at 7000 rpm for 50 min. the solution was seperated into two layers, the lower layer (red gold-labelled mAb) was collected and washed with 0.02 M phosphate buffer (containing 5% sucrose, 1% BSA and 0.5% PEG 6000, pH 7.4) by centrifuging three times to remove the unlabelled antibody. The last part was the conjugation products were reconstituted to 1 mL in 0.02 M phosphate buffer containing 0.02% NaN_3_ and then stored at 4 °C until use [18].

#### 2.3.3. Preparation of Nitrocellulose Capture Membranes

The coating antigen (CIP-BSA) and goat anti-mouse IgG were used as the capture reagent for the control line. The antigen and goal anti-mouse IgG coatings were sprayed onto the nitrocellulose (NC) membrane at 1 μL/cm using a dispenser to produce the test line and a control line on the strip. The membrane was then dried at 37 °C for 30 min and stored at room temperature until use. The capture reagent was sprayed onto a glass fiber membrane to prepare the conjugate pad, which was dried at 37 °C for 2 h. The NC membrane coated with capture reagents was pastes on the center of the plastic backing plate (polyvinylchloride (PVC)), and the conjugate pad (glass fibre), sample pad and absorbent pad were laminated and pasted onto the back plate. Finally the plate was cut into 3-mm-wide strips using a strip cutter for further detection.

#### 2.3.4. Test Procedure and Principle

The sample (80 μL) was added to the conjugate layer that contains the colloidal gold labelled antibodies. Due to capillary action, the solutions could flow in the direction of the absorbent pad. If CIP exists in the sample, it may compete with the CIP-KLH conjugates that have been embedded in the test line for the finite amount of anti-CIP mAb. Therefore, the more CIP is present in the sample, the weaker the color of the test line appears will become. When a sufficient amount of CIP is present, the free CIP may bind with all the labelled mAb, preventing any binding to the CIP-KLH on the test line. If CIP doesn't exist in the sample, the limited amount of colloidal gold-labelled mAb can be trapped by the immobilized CIP-KLH conjugations, which in turn may create a clear visible red test line. The color intensity of the test zone on the strip paper was recorded by the BioDot TSR3000 membrane strip reader at the same time (Gene Company Limited).

**Figure 2.**
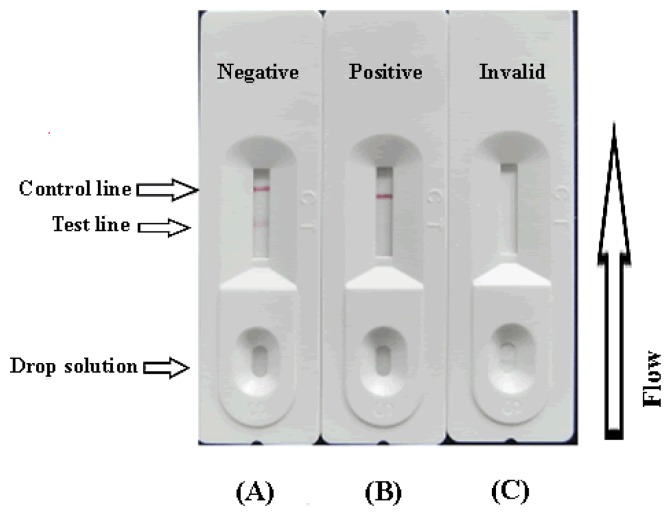
Illustration of typical strip test results. If the sample is negative (**A**). A positive result could be indicated only if the control line appears (**B**), if control line doesn't appear or only the test line appears (**C**), it suggests that the testing procedure was incorrect.

To assure that the test strip will work correctly, the flow must also reach the control line, which has goat anti-mouse IgG embedded in it, and create an indication as well. This control should always appear in a successful test, while the test line will only appear when the sample is negative (Figure 2A). A positive result could be indicated only if the control line appears (Figure 2B), if control line doesn't appear or only the test line appears (Figure 2C), which suggests that the testing procedure was incorrect or the strip was invalid, indicating that the test should be repeated using a new strip.

### 2.4. Determination of Performance

#### 2.4.1. Sensitivity of the Test Strip

The sensitivity of the test strip was determined by testing some CIP reference samples. CIP was diluted at concentrations of 0, 1, 2, 4, 5, and 10 ng/mL in 0.01 M PBS (pH 7.4) and then the detection limit was determined. The concentrations of CIP structural analogues ENR and NOR were 0, 1, 5, 6, 7, 8, and 10 ng/mL, NDF and DANO were 0, 1, 5, 6, 7, 7.5, 8, and 10 ng/mL, PEX and LOME were 0, 5, 8, 10, 15, and 25, ENO were 0, 10, 25, 40, and 50 ng/mL, and SAR were 0, 10, 25, 40, 50, 80, and 100 ng/mL, respectively. The blank sample was the dilution buffer (0.01 M PBS). Two drops (80 μL) of the solution were added on the sample pad of the strip separately with a micropipette. Three minutes later, the test strip reader recorded the color depth of the different strips. The lower detection limit (LDL) with naked eyes was defined at the amount of CIP producing a clearly visible difference in intensity of the test device in comparison with the 0 ng/mL (blank sample). Six repeat samples analyzed on a single day were tested at each concentration [24,25].

#### 2.4.2. Detection of the CIP in Milk Samples

The detection of the CIP in milk samples were determined using samples with the stated concentration in negative milk samples. The samples to be analyzed were spiked with the CIP standard solution (10 μg/mL, prepared with 0.01 M PBS, pH 7.4). The final CIP concentrations were 0.625, 1.25, 2.5, and 5 ng/mL. Six replicates were determined using the test strips.

## 3. Results and Discussion

### 3.1. Optimization of the Strip Test

**Figure 3.**
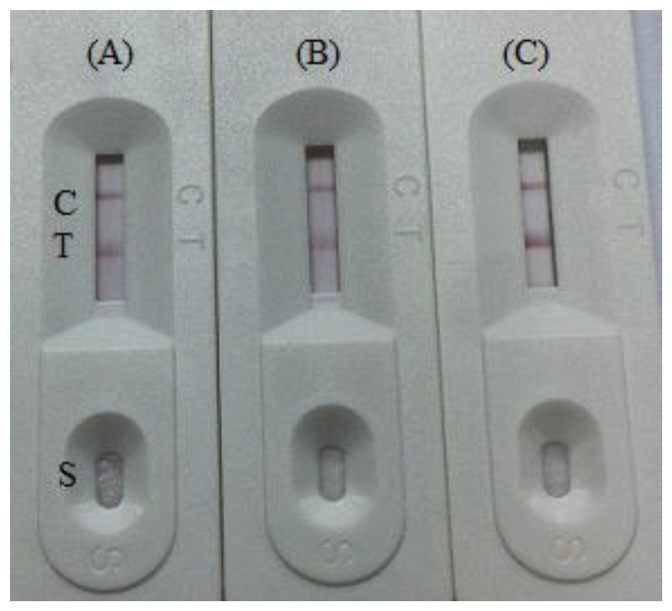
Result after optimization on pH 7 and the amount of labelled antibody using different levels (**A**) 2 μg/mL, (**B**) 4 μg/mL, and (**C**) 8 μg/mL. T, test line. C, control line. S, sample.

The amount of antibody and pH are very important during conjugation of colloidal gold with an antibody (Ab) [26]. The optimal pH and optimal amount of antibody can be determined by measuring the differential absorbance [27]. In our study, to evaluate the effect of colloidal gold particle size on the strip test sensitivity the amount of labelled antibody was optimized in these tests using different levels (2, 4, 8 μg/mL) in conjugation with 1 mL colloidal gold. In addition, different pH values (pH 7.0, 8.0, 9.2) were used for the conjugation reaction in the optimization process. It was found that the colloidal gold solution was stable at pH 7.0 and 8.0. The optimum amount of mAb was 2 μg/mL of colloidal gold (Figure 3). The time and temperature for drying the NC membrane for immobilizing the capture reagents was evaluated from 30 min to overnight at 37 °C. Capture reagent after 2 h at 37 °C demonstrated high stability and the best color [25]. In this study, we used 0.05%–1.0% casein as blocking buffer. The non-specific adsorption was not removed by the lower concentration of blocking buffer. Protein concentration (1%–3%), in general, could reduce the levels of non-specific background signal in an immunochromatographic strip (ICS) [28]. Compared with the unblocked NC membrane, the blocked membrane allows both sample solution and gold-labelled antibody to flow more slowly, and hence they are not recommended as suitable blocking materials for ICS [29].

Gold nanoparticles that range in size from 5 to 100 nm in diameter can be produced and have been conjugated successfully, but 5 nm nanoparticles do not have the bright red color, the hence give only a very weak to no signal at the detection line. Nanoparticles larger than 20 nm give a meaningful signal. In the other hand, 100 nm particles are too large compared to antibody molecules [30]. Based on previous publications from our lab [18,19,23,31], colloidal gold nanoparticles with a diameter of 30 nm were selected because of their stability, simplicity, and bright color.

**Figure 4.**
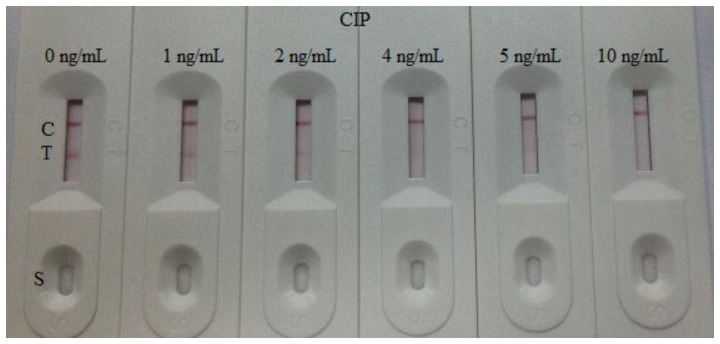
Colloidal gold immunochromatography assay for CIP. CIP concentration (from left to right): 0, 1, 2, 4, 5, 10 ng/mL. CIP, ciprofloxacin. T, test line. C, control line. S, sample.

**Figure 5.**
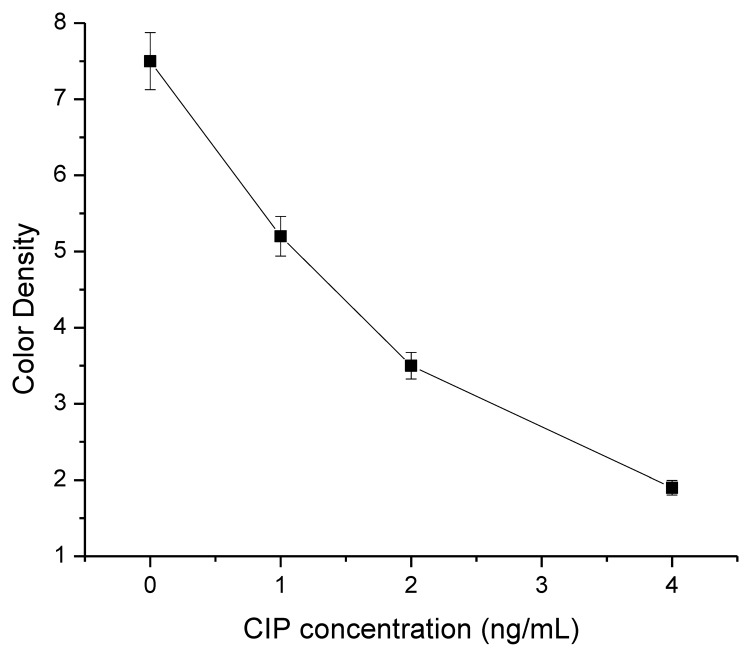
The resulting calibration curves of effect CIP concentration (0, 1, 2, and 4 ng/mL) with the color density. Each sample was analyzed for six replicates and error bars represent the standard deviations.

The assay sensitivity was investigated with a series of diluted CIP standards. The LDL with the naked eye was set at the amount of CIP producing a clearly visible difference in intensity of the test strip in comparison with that when no CIP was added in the sample as a negative control. As shown in Figure 4, the signal color on the test lines changed from strong to weak and disappeared completely at 5 ng/mL. Under the optimized detection conditions, a scanning reader was used to measure the intensity of the signal on the test zone and the corresponding optical responses curves are shown in Figure 5.

### 3.2. Specificity of the Strip Test

**Figure 6.**
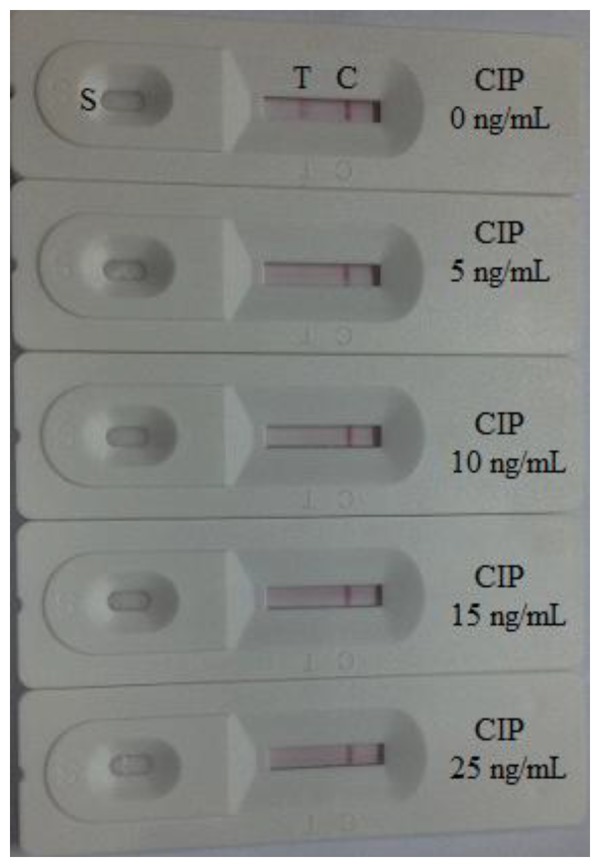

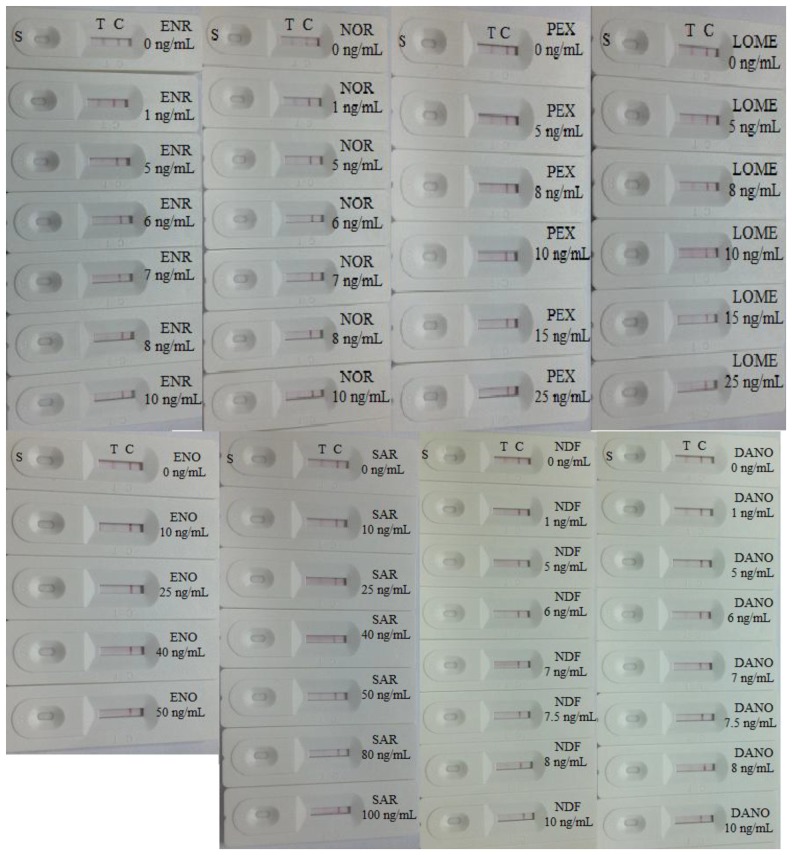
The cross-reaction of the CIP test strip with ENR, NOR, NDF, DANO, PEX, LOME, ENO, and SAR. ENR and NOR concentrations: 0, 1, 5, 6, 7, 8, and 10 ng/mL. NDF and DANO concentrations: 0, 1, 5, 6, 7, 7.5, 8, and 10 ng/mL. PEX and LOME concentrations: 0, 5, 8, 10, 15, and 25 ng/mL. ENO concentration: 0, 10, 25, 40, 50. SARA concentration: 0, 10, 25, 40, 50, 80, and 100 ng/mL. ENR, enrofloxacin; NOR, norfloxacin; NDF, nadifloxacin; DANO, danofloxacin; PEX, pefloxacin; LOME, lomefloxacin; ENO, enoxacin; SAR, sarafloxacin. T, test line. C, control line. S, sample.

The cross-reaction of the assay was determined by running ENR and NOR at concentrations of 0, 1, 5, 6, 7, 8 and 10 ng/mL, NDF and DANO at concentrations of 0, 1, 5, 6, 7, 7.5, 8, and 10 ng/mL, PEX and LOME at concentrations of 0, 5, 8, 10, 15, and 25 ng/mL, ENO at concentrations of 0, 10, 25, 40, 50 ng/mL and SAR at concentrations of 0, 10, 25, 40, 50, 80, 100 ng/mL. As shown in Figure 6, there was no color at the test line position in the different CIP strips expect in the 0 ng/mL strip. Nevertheless, the ENR and NOR samples presented a weak color in all test strips, a strong color at 0 ng/mL and no color at 7 ng/mL. The NDF and DANO samples presented a weak color at 1, 5, and 6 ng/mL and no color at 7.5 ng/mL (NDF) and 10 ng/mL (DANO), respectively. Meanwhile, the PEX and LOME samples also presented strong color at 0, 5, and 8 ng/mL, weak color at 10 ng/mL and no color at 15 ng/mL (PEX) and 25 ng/mL (LOME). The ENO sample showed a weak color at 10 and 25 ng/mL and no color at 40 ng/mL. On other hand, the SAR sample presented a good color at 10, 25, and 40 ng/mL, weak color at 50 ng/mL and no color at 80 ng/mL. Thus, the cross-reactivities (cross-reactivity = the cut-off limit of CIP/the cut off limit of structural analogues) of the CIP test strip to ENR, NOR, NDP, DANO, PEX, LOME, ENO, and SAR were 71,4%, 71.4%, 66%, 50%, 33%, 20%, 12.5%, and 6.25%, respectively. As shown in Figures 4 and 6, the red-purple color intensities were similar on the control lines of the different strips, indicating the validity of the assay.

Even though a previous publication [4] has reported a CIP test using an ELISA assay and the result was more sensitive that reported herein, that detection process was still time consuming and involved multiple incubation and wash steps. Due to its speed, simplicity, and low-cost characteristics, the immunochromatograhic strip proposed in this study can detect eight different kind of FQs.

### 3.3. Detection of the CIP in Milk Samples

The detection of the CIP in milk samples were performed using samples at the stated concentration in negative milk samples. The samples to be analyzed were spiked with the CIP standard solution (10 μg/mL, prepared with 0.01 M PBS, pH 7.4). The final CIP concentrations were 0.625, 1.25, 2.5, and 5 ng/mL. Six replicates were determined using test strips. In this study, the matrix effect was determined for milk samples. One of the major advantages of an immunochromatograhic strip (ICS) assay is that it is a very simple and rapid test. Samples were prepared by performing a simple dilution step with the sample diluent. Negative samples were spiked with different levels of CIP to determine the detection limit of the strip test. The test line can be evaluated directly by visual assessment. If the color of the test line was similar to that of the negative control sample, the sample was considered to be negative. In the other hand, if the color of the test line was between that of the two definite control samples, the CIP concentration was considered to lie between the two control samples. When the concentration of the CIP residue was greater then there was essentially no color observable at the test line, the sample should be judged as overtested. In this study, the detection limit of the strip test was defined as the CIP concentration of the test line.

**Figure 7.**
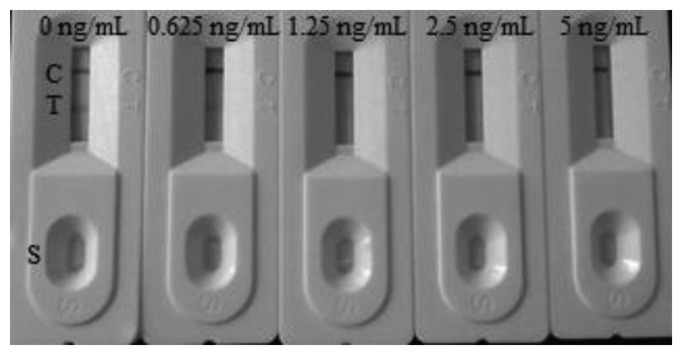
Result of CIP detection via the colloidal gold immunochromatographic strip assay (ng/mL) in spiked milk (n = 6). T, test line. C, control line. S, sample.

The samples were spiked with the CIP standard solution (10 μg/mL, prepared with 0.01 M PBS, pH 7.4). The final milk samples were spiked with CIP at 0, 0.625, 1.25, 2.5, and 5 ng/mL (n = 6). The spiked series were prepared and analyzed with the optimal test strips. The color intensity decreased with increasing CIP concentration. The results are summarized in Figure 7. The signal color on the test lines changed from strong to weak and disappeared completely at 2.5 ng/mL. The MRL has been set by the European Commission at 100 ng/mL for the enrofloxacin and ciprofloxacin in milk. The results of this study demonstrate the lowest detection limit of the immunochromatography assay is >2.5 ng/mL, and drug concentrations lower than this limit cannot be recognized.

To determine the accuracy, milk samples containing 0.625 and 1.25 ng/mL of CIP were tested. The test was carried out on six replicates with a single batch of test strips and the result of the test measured using the test strip reader. The highest relative standard deviation was 8.66%. The results are shown in Table 1.

## 4. Conclusions

A sensitive colloidal gold-based lateral-flow immunochromatography assay was developed for the rapid detection of CIP in milk samples. A highly sensitive and specific anti-CIP antibody was obtained from BALB/c mice with CIP-KLH conjugates. Our results demonstrated that the cut-off limits of the semi-quantitative test strip for CIP can be as low as 5 ng/mL in PBS and 2.5 ng/mL in milk samples and the test had cross-reactivity with eight different kind of fluoroquinolones.

Because of its rapid and simple performance with high throughput and sensitivity, the colloidal gold-based immunochromatographic strip test can be applied to the detection of CIP. This method also allowed the detection of CIP at low concentration levels and permitted multiple dilutions of high sample concentrations, which is useful for research and practical purposes. The CIP strip test can be completed within 3 min in only one step and with an easy procedure and equipment. As this method provides only preliminary qualitative results, determined positive samples should be further confirmed by some more accurate and quantitative method such as HPLC, LC-MS/MS or an ELISA method.

## Figures and Tables

**Table 1.** Analysis of milk samples fortified with ciprofloxacin (CIP).

Spiked CIP (ng/mL)	Mean ± SD (ng/mL, n = 6)	CV (%)
0.625	0.59 ± 0.12	8.66
1.25	0.97 ± 0.06	5.97
